# Maternal iron levels early in pregnancy are not associated with offspring IQ score at age 8, findings from a Mendelian randomization study

**DOI:** 10.1038/ejcn.2013.265

**Published:** 2014-01-08

**Authors:** S J Lewis, C Bonilla, M-J Brion, D A Lawlor, D Gunnell, Y Ben-Shlomo, A Ness, G D Smith

**Affiliations:** 1 School of Social and Community Medicine, University of Bristol, Bristol, UK; 2 MRC Integrative Epidemiology Unit, School of Social and Community Medicine, University of Bristol, Bristol, UK; 3 School of Oral and Dental Sciences, University of Bristol, Bristol, UK

**Keywords:** iron, IQ, intelligence, genetic variants, mendelian randomization, ALSPAC

## Abstract

**Background/Objectives:**

Iron is fundamental to many basic biological functions, and animal studies suggest that iron deficiency early in life can have a lasting impact on the developing brain.

**Subjects/Methods:**

We used a population-based cohort of mothers and their children to assess the effect of iron status among pregnant women on the cognitive ability of their offspring. But to avoid the inherent confounding that occurs within observational epidemiology studies we examined the association of maternal genotype at single-nucleotide polymorphisms in the genes *HFE* (rs1799945) and (rs1800562), *TF* (rs3811647) and *TMPRSS6* (rs1800562), which are related to iron, haemoglobin or transferrin levels, on their child's cognitive test scores at age 8.

**Results:**

We found strong associations between *HFE* and *TMPRSS6* genotypes and mother's haemoglobin levels early in pregnancy (*P*-values are all ⩽4.1 × 10^−5^) and a genetic score comprised of alleles at these loci was even more strongly associated with haemoglobin levels (*P*=3.0 × 10^−18^), suggesting that this was a good instrument to use to look at the effect of prenatal iron levels on offspring cognition. However, mother's genotype at the above loci was not associated with offspring IQ at age 8.

**Conclusions:**

We therefore concluded that there is no evidence of an effect of exposure to low levels of iron (within the normal range) in pregnancy on offspring cognition at age 8. However, pregnant women in the UK with low haemoglobin levels are prescribed iron supplements and so we were unable to look at the effect of iron deficiency in our study.

## Introduction

Iron is fundamental to many basic biological functions including oxygen transport, production of adenosine triphosphate (ATP), DNA synthesis, mitochondrial function and protection of cells from oxidative damage.^1^ However, iron deficiency is the most common nutritional deficiency worldwide, with around 1.6 billion people thought to be affected. This is particularly the case in developing countries where 25% of pregnant women are reported to be iron deficient.^2^

Animal studies suggest that iron deficiency early in life can have a lasting impact on the developing brain.^3^ In humans, the brain is likely to be most vulnerable to nutrient deficiencies during the brain growth spurt that occurs in the period between the last trimester of fetal life and the first 2 years of childhood.^3^

A recent meta-analysis of five randomized controlled trials of early life iron supplementation on motor and mental development in children under 3 years of age found evidence of a beneficial effect on motor development and very weak evidence of a beneficial effect on mental development.^4^ The same review found only one randomized controlled trial that specifically addressed the effect of prenatal iron supplementation on child's IQ (at age 4). This study was carried out in Australia, and showed no differences in IQ between the two groups. There was, however, a very high drop-out rate (30%), so the sample used for the final analysis may not be representative of those randomized.^5^ Further intervention studies of iron supplementation among pregnant women would need robust evidence to justify their conduct, as around half of all pregnant women require iron supplements to prevent anaemia when pregnant. Although observational studies are feasible, they are limited in their ability to examine whether maternal iron status in pregnancy affects offspring neurological development and cognitive function, because they are likely to be confounded by socio-economic background, and related lifestyle factors, which are strongly associated with both nutritional status^6^ and childhood cognitive function.^7^

Associations between genetic polymorphisms, which are associated with iron levels, and cognition are less likely to be subject to the problem of confounding by lifestyle factors, which occurs in observational studies,^8^ and studies of this type are not subject to the ethical issues that arise in randomized controlled trials.^9^ We used genetic variants known to be associated with iron levels to determine whether maternal iron status during pregnancy was causally associated with child's IQ score at age 8 in a large population-based study.

## Materials and methods

### Study population

**Figure 1:**
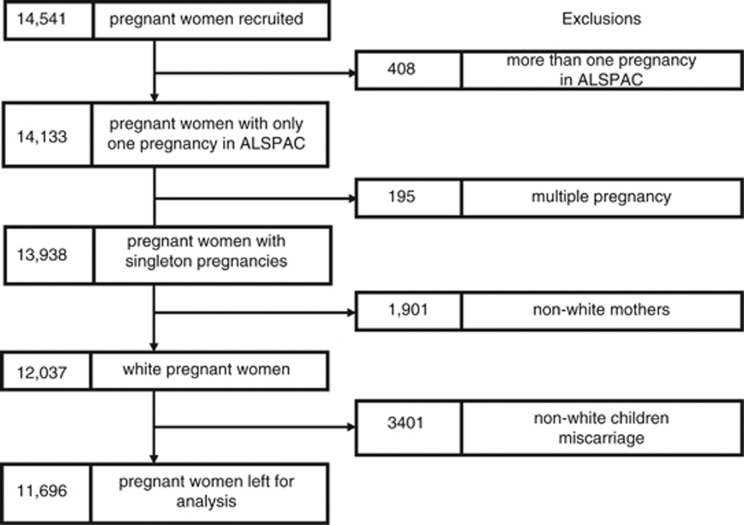
Flow diagram showing total number of women recruited into the study with reasons for exclusion and numbers excluded at each stage.

The Avon Longitudinal Study of Parents and Children (ALSPAC) is a population-based prospective study investigating factors that affect the health and development of children and their parents. The study methods are described in detail elsewhere (http://www.alspac.bris.ac.uk^10, 11, 12^). In brief, pregnant women living in Bristol, UK who had an expected date of delivery between April 1991 and December 1992 were eligible. A total of 14 541 pregnant women enrolled in the study. A detailed outline of the exclusion criteria for this analysis and numbers with missing data is given in Figure 1. Extensive data have been collected from the mothers from pregnancy onwards by questionnaire, abstraction from medical notes, record linkage and by attendance at research clinics. Ethical approval for the study was obtained from the ALSPAC law and ethics committee and the local research ethics committees.

### Ethnicity

We excluded all non-white women from this analysis to avoid population stratification (see Figure 1 for exclusions). Ethnicity was available from self-report or had been imputed from five genetic ancestry-informative markers with established population-specific allelic distributions: rs713598 and rs1726866 in TAS2R38,^13^ rs4988235 in MCM6,^14^ rs41310927 in ASPM^15^ and rs930557 in MCPH1.^15^

### Measurement of cognition

Cognitive testing of children was carried-out during a clinical visit when the children were aged 8 using a shortened version of the Wechsler Intelligence Scale for Children (WISC-III), described in detail elsewhere.^16^ An overall age-adjusted cognitive score was derived from this assessment for each child who completed the test.^17^ This test has been shown to be associated with mother's educational level, parity, socio-economic status and several other socio-economic and lifestyle factors in a previous analysis, thus we are confident of the validity of this test (supplementary tables in Bonilla *et al.*^18^).

### Measurement of mothers educational level

Mother's educational level was obtained by questionnaires administered to the mother during pregnancy, in which women were asked to report their highest educational attainment from five-ordered categories. For the purposes of adjustment in the models of mother's genotype and child's IQ, the five categories were used. However, when mother's education was used as the outcome, these categories were collapsed as follows: ordinary level (O-level) or equivalent vs higher or lower. The O-level was an exam-based qualification for students aged 14–16 years (16 is the legal minimum school leaving age in the UK), which was replaced by the General Certificate of Secondary Education (GCSE) in 1988 in the UK. Further details are available at http://www.direct.gov.uk/en/EducationAndLearning/QualificationsExplained/DG_10039024.

### Iron supplementation

ALSPAC mothers were asked at 18 and 32 weeks of pregnancy whether they had taken iron supplements during this pregnancy, they were also asked to list any medications they were taking as a text answer. If mothers reported taking iron supplements and/or taking medications or supplements containing iron, they were classed as taking iron supplements. We constructed three groups as follows: (1) not taking iron supplements, women who did not report taking supplements or medication containing iron at 18 or 32 weeks of pregnancy; (2) taking iron supplements at 18 weeks, women who reported taking supplements or medication containing iron at 18 weeks of pregnancy, irrespective of whether they were taking supplements at 32 weeks or not; (3) taking iron supplements at 32 weeks, women who reported taking supplements or medication containing iron at 32 weeks but not at 18 weeks of pregnancy.

### Measurement of confounders

Data on selected characteristics from ALSPAC mothers were used to conduct an assessment of the potential for confounding of the genotype–outcome association. These were collected either from hospital records or from the questionnaires completed by the mother during pregnancy. Mother's social class was based on occupation and determined according to the 1991 British Office of Population Statistics classification. Marital status, housing tenure, parity, inter-pregnancy interval, breastfeeding, infection during pregnancy, ever smoked, alcohol consumption before supplements during pregnancy, calcium supplements during pregnancy, folate supplements during pregnancy, other supplements and sex of the child were all tested as potential confounders.

### Laboratory methods

DNA was extracted using the salting out method. This was part of a larger study in which 58 single-nucleotide polymorphisms (SNPs) related to nutrient intake or levels were genotyped to look at the effect of early life nutrition on child's IQ score. In addition, a genome-wide association study was later carried out in this cohort. Four SNPs previously shown to cause haemochromatosis^19^ or to be strongly associated (for all *P*<4 × 10^−6^) with serum iron, serum transferrin or haemoglobin^20^ were genotyped, these were *HFE* rs1800562, rs1799945; *TF* rs3811647; and *TMPRSS6* rs4820268. A summary of what is known about these SNPs is given in Text Box 1. Genotyping was undertaken by KBioscience Ltd (www.kbioscience.co.uk), who use their own form of competitive allele-specific PCR system (KASPar) and Taqman for SNP analysis. KBiosciences have their own quality control checks. They state that to deem an assay successful it must possess three distinct clusters: the water controls must be negative, the number of genotypes callable must be >90% and the minor allele frequency should be >2%. In addition, we added blind duplicates to the samples, we checked that there were in fact three true clusters for each genotype, and where there was evidence that an SNP was not in Hardy–Weinberg equilibrium, we looked at the clusters by eye to verify whether there was any evidence of miscalling of genotypes or whether any of the unresolved genotypes could be called.

### Diplotypes and genotype score

As haemochromatosis can arise from having a rare homozygous genotype at rs1799945 or rs1800562 in the *HFE* gene or by being a composite heterozygote with one rare allele at each site, we combined genotypes at these two loci as follows: two low-iron alleles (any two haemochromatosis alleles); three low-iron alleles (heterozygotes at just one locus, with the common homozygous genotype at the other locus); four low-iron alleles (double homozygotes for common alleles).

Genotype score comprised alleles at three loci, the two above in *HFE* and rs4820268 in*TMPRSS6*, with risk alleles defined as those associated with lower haemoglobin levels, that is, allele C at rs1799945, G at rs1800562 and G at rs4820268. Individuals were categorised according to whether they had 2 or 3, 4, 5 or 6 risk alleles. No individual had 0 or 1 low iron allele.

### Measurement of haemaglobin in ALSPAC

Mother's haemoglobin levels were measured routinely at various time points during pregnancy by healthcare workers and were extracted from obstetric notes. The levels used to assess associations with outcome and genotype were the first measurement taken during pregnancy, with most (87%) taken before 18 weeks of pregnancy, and the last measurement taken during pregnancy (for this we specified that the measurement should have been done after 28 weeks of pregnancy).

### Statistical analysis

Ordered logistic regression analysis was carried out in Stata (Stata Corporation, College Station, TX, USA) to test for associations of mother's educational level (<O-level, O-level, >O-level) with genotype without adjustment and with adjustment for population stratification. Associations between supplement use (not taking supplements, taking supplements at 18 weeks and taking supplements at 32 weeks only) and genotype, and categorical confounders and genotype were tested using *χ*^2^-tests. Linear regression models were used to examine the association of maternal genotype (exposure) with WISC score or haemoglobin as outcome, and to test the association between mother's haemoglobin level and child's WISC score. Per allele odds ratios are reported for individual genotypes/genotype scores.

We carried out crude analyses of mother's genotype and child's WISC score, we also carried out an analysis adjusting for: child's genotype (to assess whether mother's genotype was exerting an independent effect on outcome rather than affecting outcome by influencing child's genotype); mother's educational level (there were two reasons for adjusting for this—first, in a sensitivity analysis where we adjusted for all potential confounders, only mother's educational level exerted an independent effect on outcome and second, we wanted to exclude the possibility that mother's genotype was influencing outcome by affecting her own educational attainment); we also adjusted for population stratification using the top 10 principal components (PCs), from genome wide association data available in ALSPAC, that reflect the population's genetic structure estimated according to Price *et al.*^21^ Assumptions of Hardy–Weinberg equilibrium were formally tested using a likelihood ratio test and the asymptotic *P*-value is reported. All the above analysis was carried-out using Stata 11.0.

## Results

The *TMPRSS6* rs4820268 polymorphism showed evidence of Hardy–Weinberg disequilibrium in the mothers (*P*=0.001) but not in the children in ALSPAC (Table 1), this was due to a slight excess in heterozygotes compared with the number expected. We studied the raw data, but could find no evidence of miscalling or that homozygotes were over-represented among the women for whom genotype was not resolved. All other genotypes were in accordance with Hardy–Weinberg equilibrium in ALSPAC. Therefore no genotype was excluded on the basis of Hardy–Weinberg equilibrium.

### Mother's haemoglobin levels by genotype in early pregnancy

We found a strong association between *HFE* genotypes and haemoglobin levels measured early in pregnancy (Table 2) and similar effects with haemoglobin measured late in pregnancy (results not shown), with rare homozygotes having higher levels compared with wild-type homozygotes and levels among heterozygotes being intermediate between the two. *TMPRSS6* rs4820268 was also strongly associated with haemoglobin levels at both time points with the rare homozygote GG having the lowest levels. The *TF* SNP investigated in this study did not show evidence of being associated with haemoglobin levels, for this reason this SNP was not included in the genotype score. Both the *HFE* diplotype and the genotype score based on both *HFE* genotypes and *TMPRSS6* rs4820268 explained more of the variation in haemoglobin levels than individual SNPs alone, with the genotype score showing very strong evidence of a dose–response relationship with haemoglobin levels, but still only explaining 1.02% of the variation in early pregnancy haemoglobin levels.

### Genotypes and iron supplementation during pregnancy among ALSPAC mothers

Table 3 shows associations between taking iron supplements during pregnancy and genotype. Both the *HFE* loci were associated with taking supplements during pregnancy such that women with rare homozygote genotypes were less likely to take supplements, with some evidence of a dose response (albeit weak evidence). Women carrying the *TMPRSS6* rs4820268 G allele were more likely to take supplements during pregnancy, and there seemed to be a per allele effect for this genotype. The *TF* rs3811647 SNP showed no clear pattern of association with taking supplements. Although there was weak evidence that women with the heterozygous genotype were less likely to take supplements. Furthermore, *HFE* diplotypes and a genotypic score containing both *HFE* SNPs and *TMPRSS6* rs4820268 were stronger predictors of supplement use than individual genotypes.

### Genotypes and potential confounders

We looked at the association between genotype/diplotype/genotype score in mothers and 19 potential confounding factors. We found evidence for a small number of associations at *P*=0.05 level, which are as follows: *HFE* rs1800562 and alcohol intake before pregnancy (*P*=0.02), *HFE* rs1800562 and social class (*P*=0.02), *HFE* rs1800562 and taking folate supplementation (*P*=0.001), *TMPRSS6* rs4820268 and parity (*P*=0.01), These four associations are 5.3% of the total number of genotype–potential confounder associations tested (that is, the expected number (5%) due to chance). Associations between confounders and diplotypes/genotype score largely reflected associations with individual genotypes and were as follows: *HFE* diplotypes and social class (*P*=0.001); *HFE* diplotypes and folate supplementation (*P*=0.001); genotype score and infection during pregnancy (*P*=0.003); and genotype score and folate supplementation (*P*=0.001).

### Genotype and mothers' educational level

We found associations between mothers' educational level and *HFE* rs1799945 genotypes, such that mothers with the rare allele (associated with higher iron levels) were more likely to have an educational level that was greater than O-level (Table 4). We also found an association between the *TF* rs3811647 genotype and mother's educational level, such that the A allele (associated with higher transferrin levels) was associated with a higher educational attainment. Adjustment for population stratification did not change these results.

### Mother's haemoglobin level in pregnancy and child's IQ

Mother's haemoglobin levels early in pregnancy among mothers on whom we had offspring IQ data were on average (mean) 12.3 g/dl, with a range of 7.2–16.2, and only 7.8% of mothers had levels considered to be low (<11 g/dl). If we take the last haemoglobin measurement that was available during pregnancy, the mean haemoglobin level during pregnancy among mothers who had children with WISC data was 11.4 g/dl, range 7.3–15.7, with 30% of mothers having haemoglobin levels of <11 g/dl. Mothers' haemoglobin level (g/dl) early in pregnancy (adjusted by gestation in days when measured) was not found to be associated with child's WISC score at age 8 (mean difference in WISC score per g/dl=0.01 (95% confidence interval (CI)=−0.44 to 0.46), *P*=0.96) even after the adjustment for iron supplement intake during pregnancy (mean difference=0.11 (95% CI=−0.37 to 0.60) *P*=0.65). There was weak evidence that haemoglobin levels measured after 28 weeks of pregnancy (adjusted by gestation in days when measured) were associated with the child's IQ score (mean difference in WISC score per g/dl=0.41 (95% CI=−0.07 to 0.46), *P*=0.09), but this effect disappeared after adjustment for potential confounders g/dl=0.01 (95% CI=−0.53 to 0.51), *P*=0.98).

### Mother's genotype and child's IQ score

We found no evidence of associations between mother's genotype and child's WISC score at age 8 (Table 5) in our crude analysis and in an analysis adjusted by iron supplementation, child's genotype, mother's education level and population stratification.

## Discussion

We found strong evidence of associations between two SNPs in *HFE*, which have been previously shown to cause iron overload, and haemoglobin levels during early pregnancy in this large population based study of women. We also found strong associations between rs4820268 in *TMPRSS6* and haemoglobin levels. In addition, an iron genotype score that comprised of the above three SNPs was associated (*P*=1.9 × 10^−18^) with a 0.3 s.d. difference in haemoglobin levels. Therefore, we concluded that we had a good genetic instrument with which to determine whether iron levels during pregnancy were associated with offspring cognition. We did not find any evidence for an association between *TF* rs3811647 and haemoglobin levels, although the A allele at this site has previously been reported to be associated with serum transferrin and reduced transferrin saturation,^20^ it has not previously been demonstrated to be associated with haemoglobin to our knowledge.

The association between mother's genotype and haemoglobin was strong at the beginning of pregnancy, and we expected this association to be less evident later on in pregnancy, as women in the UK with low iron levels during pregnancy are advised by a medical practitioner to take supplements. Those genotypes associated with low haemoglobin levels are also associated with an increased likelihood of receiving iron supplements, and supplementation will remove or at least diminish an association between genotype and iron levels. However, we tested by comparing genotypes with haemoglobin later in pregnancy and found that the associations were as strong.

We found no evidence that low iron levels in pregnancy have a detrimental effect on the developing fetus' brain, either in the observational analysis (using haemoglobin measured in early and late pregnancy) or using genotypes in the Mendelian randomization approach. Our study had sufficient power to detect a 0.80-unit change in IQ score per g/dl increase in haeomglobin with a *P*-value <0.05 (assuming 80% power) in the observational analysis. As a 1-g/dl change in haemoglobin levels is quite large, this is likely to be at the lower end of what is clinically relevant, thus we were satisfied that we had sufficient power to detect an effect in the observational analysis. The power to detect an effect in the Mendelian randomization analysis is much lower; however, as we did not see an effect of haemoglobin in the observational analysis, we are not concerned that we missed an important effect of prenatal exposure to iron on childhood IQ due to low power in the study. There are several limitations to this analysis, which are described below. The first is that while nutrient intake and therefore haemoglobin levels of the mother during pregnancy are expected to be strongly confounded by socio-economic factors, the expectation is that genotype will not be associated with these factors.^8^ In this study, we found some evidence that rare alleles in *HFE* rs1800562 and *TF* rs3811647 were associated with an increased educational attainment among ALSPAC mothers. These variants are both likely to have an effect on overall iron availability. The rare variant in *HFE* rs1800562 causes iron overload and is associated with an increase in haemoglobin levels,^19, 22^ whereas the rare allele in *TF* rs3811647 is associated with higher transferrin levels^20^ and lower transferrin saturation.^20^ But more importantly for this study, as maternal educational level is one of the strongest predictors of childhood cognition, this violates the assumption of no confounding by genotype. However, adjustment of our mothers' genotype–childs' IQ analysis by mother's educational level did not change our conclusions. The frequency of the alleles used in this analysis differs widely by population, so there is also the potential for any association to be confounded by population structure. However, we adjusted our analysis by the top 10 principal components that reflect the population structure from GWAS data carried out on this population and found that this adjustment did not affect our conclusions.

The *HFE* variant rs1800562 that was associated with educational status was also associated with social class, which may be a chance finding or may be a consequence of the association with educational status, as the two are closely linked. Similarly, the association between genotypes at this loci and taking folic acid supplements could be due to chance or could be because many women taking iron supplements may take iron with folic acid.

A further limitation of this study is that the number of women with *HFE* rare homozygous genotypes were small and our study therefore does not have any power to detect effects with these genotypes. Replication in a larger study would be desirable to confirm our findings; however, we are not aware of any large studies with DNA from mothers and IQ from children that could readily do this analysis.

Finally, as this is a relatively well-nourished population who were closely monitored during pregnancy, and given supplements if their iron levels were found to be low, it is possible that the haemoglobin levels in this study simply did not fall low enough to be detrimental to the fetus. By the end of pregnancy 30% of the mothers had levels that are generally considered to be low. However, it is not known whether these are sufficiently low as to affect cognitive development in their offspring, indeed it is likely that levels in the mothers are low, because iron is preferentially diverted to the fetus to negate any deleterious effects.

In conclusion, our results do not support the hypothesis that exposure to low levels of iron early in fetal life adversely affect brain development and therefore IQ in childhood. However, our findings should be interpreted with caution owing to the fact that pregnant women with low haemoglobin levels in the UK receive iron supplements fairly early on in pregnancy; thus, we are unable to study the effects of low iron in the second and third trimester of pregnancy in this cohort.

## Figures and Tables

**Table 1:** Hardy-Weinberg equilibrium test for mothers' and children's genotypes

*dbSNP*	*Major/minor alleles*	*Mothers*		*Children*	
		P*-value*	N	P*-value*	N
rs1799945	C/G	0.20	6637	0.08	8294
rs1800562	G/A	0.74	6627	0.15	8227
rs3811647	G/A	0.44	6594	0.14	8266
rs4820268	A/G	0.001	6616	0.08	8289

**Table 2:** Maternal genotypes at SNPs in iron-related genes and Hb levels measured early in pregnancy adjusted by gestational age at the time of measurement

*Genotype*	*Mean*	*s.d*	N	*Coefficient*	*s.e*	*Adj* P*-value*	*% Variance explained*
*rs1799945 HFE*							
GG	12.5	0.9	144	–0.093	0.023	4.1 × 10^−5^	0.19
CG	12.4	1.0	1532				
CC	12.3	1.0	4731				
							
*rs1800562 HFE*							
AA	12.6	1.1	29	–0.174	0.032	4.6 × 10^−8^	0.34
GA	12.5	1.0	828				
GG	12.3	1.0	5541				
							
*rs3811647 TF*							
GG	12.3	1.0	2766	–0.007	0.017	0.69	0.00
GA	12.3	1.0	2885				
AA	12.3	0.9	717				
							
*rs4820268 TMPRSS6*							
AA	12.4	1.0	1721	–0.103	0.016	3.6 × 10^−10^	0.55
AG	12.3	1.0	3316				
GG	12.2	1.0	1351				
							
*rs1799945/rs1800562*							
2 risk alleles	12.6	1.0	297	–0.136	0.020	4.9 × 10^−12^	0.56
3 risk alleles	12.4	1.0	2080				
4 risk alleles	12.3	1.0	3931				
							
*Genotypic score*							
⩽3 risk alleles	12.5	1.0	822	–0.113	0.013	3.0 × 10^−18^	1.02
4 risk alleles	12.4	1.0	2093				
5 risk alleles	12.3	1.0	2462				
6 risk alleles	12.2	1.0	844				

**Table 3:** Proportion of mothers taking iron supplements at 18 or 32 weeks of pregnancy according to their genotype at iron-related SNPs

*dbSNP*	*Gene*	*Genotype*	*Not taking supplements (%)*	*Taking supplements at 18 weeks (%)*	*Taking supplements at 32 weeks (%)*	P*-value*
rs1799945	HFE	GG	63 (48.8)	42 (32.6)	24 (18.6)	0.11
		CG	676 (46.7)	433 (29.9)	339 (23.4)	
		CC	1927 (43.4)	1390 (31.3)	1123 (25.3)	
rs1800562	HFE	AA	17 (65.4)	5 (19.2)	4 (15.4)	0.04
		GA	367 (47.9)	230 (30.0)	169 (22.1)	
		GG	2285 (43.8)	1623 (31.1)	1307 (25.1)	
rs3811647	TF	GG	1117 (43.2)	787 (30.4)	681 (26.3)	0.04
		GA	1244 (45.7)	851 (31.3)	624 (23.0)	
		AA	283 (42.0)	218 (32.3)	173 (25.7)	
rs4820268	TMPRSS6	AA	759 (47.4)	481 (30.1)	360 (22.5)	0.01
		AG	1378 (44.1)	969 (31.0)	780 (24.9)	
		GG	521 (40.9)	415 (32.6)	339 (26.5)	
rs1799945/rs1800562	2 risk alleles	0	144 (53.1)	77 (28.4)	50 (18.5)	0.001
	3 risk alleles	1	907 (46.4)	591 (30.3)	455 (23.3)	
	4 risk alleles	2	1580 (42.7)	1164 (31.5)	955 (25.8)	
genotypic score	⩽3 risk alleles	0	391 (51.4)	217 (28.6)	152 (20.0)	<0.001 X2=29.66 6 df
	4 risk alleles	1	897 (45.7)	608 (31.0)	456 (23.3)	
	5 risk alleles	2	982 (42.2)	725 (31.1)	621 (26.7)	
	6 risk alleles	3	325 (40.8)	262 (32.9)	210 (26.3)	

**Table 4:** Maternal education levels according to genotype at iron-related SNPs

*dbSNP*	*Gene*	*Genotype*^a^	*<O level (%)*	*O level (%)*	*>O level (%)*	*Unadjusted OR (95% CI) P-value* N	*Adjusted*^b^ *OR (95% CI) P-value* N
rs1799945	HFE	GG	30 (22.4)	40 (29.8)	64 (47.8)	0.89 (0.81, 0.98) 0.01 6152	0.89 (0.80, 0.99) 0.03 4535
		CG	418 (28.3)	540 (36.6)	518 (35.1)		
		CC	1378 (30.4)	1600 (35.2)	1564 (34.4)		
rs1800562	HFE	AA	8 (28.6)	9 (32.1)	11 (39.3)	1.00 (0.88, 1.14) 1.00 6139	0.99 (0.85, 1.15) 0.89 4521
		GA	259 (32.7)	237 (29.9)	296 (37.4)		
		GG	1553 (29.2)	1928 (36.3)	1838 (34.5)		
rs3811647	TF	GG	803 (30.1)	954 (35.8)	905 (34.0)	1.08 (1.01, 1.16) 0.03 6112	1.10 (1.01, 1.19) 0.03 4517
		GA	829 (29.9)	971 (35.0)	971 (35.0)		
		AA	184 (27.1)	226 (33.3)	269 (39.6)		
rs4820268	TMPRSS6	AA	506 (30.9)	567 (34.6)	566 (34.5)	1.03 (0.97, 1.10) 0.34 6133	1.03 (0.95, 1.11) 0.45 4533
		AG	924 (29.0)	1153 (36.1)	1115 (34.9)		
		GG	383 (29.4)	453 (34.8)	466 (35.8)		
rs1799945/rs1800562	HFE	2 risk alleles	81 (28.8)	80 (28.5)	120 (42.7)	0.92 (0.85, 0.99) 0.04 6055	0.91 (0.83, 1.00) 0.05 4465
		3 risk alleles	581 (29.1)	708 (35.4)	710 (35.5)		
		4 risk alleles	1135 (30.1)	1359 (36.0)	1281 (33.9)		
genotypic score	HFE-TMPRSS6	⩽3 risk alleles	241 (30.6)	249 (31.9)	294 (37.5)	0.99 (0.94, 1.04) 0.61 5974	0.98 (0.92, 1.04) 0.54 4417
		4 risk alleles	591 (29.5)	724 (36.2)	687 (34.3)		
		5 risk alleles	684 (28.8)	872 (36.7)	818 (34.5)		
		6 risk alleles	252 (30.9)	279 (34.3)	283 (34.8)		

a Genotypes are ordered from higher to lower associated iron levels.

b OR obtained using ordered logistic regression of 3-level maternal education and genotype, adjusted by maternal principle component.

**Table 5:** Maternal genotypes at SNPs in iron-related genes and full scale IQ of their children at 8 years of age

*dbSNP*	*Gene*	*Genotype*	*Full scale IQ*	*s.d*	N	*Unadjusted*^a^ *effect (95% CI) P-value* N	*Adjusted*^a^^b^ *effect (95% CI) P-value* N
rs1799945	HFE	GG	105.9	17.0	79	0.50 (–0.59, 1.58) 0.37 3543	1.09 (–0.27, 2.45) 0.39 2402
		CG	102.9	16.3	850		
		CC	104.1	16.4	2614		
rs1800562	HFE	AA	107.1	12.8	15	–0.52 (–2.05, 1.00) 0.50 3535	0.38 (–1.57, 2.32) 0.71 2352
		GA	104.2	16.6	455		
		GG	103.8	16.5	3065		
rs3811647	TF	GG	104.0	16.3	1511	–0.13 (–0.95, 0.69) 0.76 3514	–0.36 (–1.40, 0.68) 0.50 2378
		GA	103.9	16.3	1613		
		AA	103.6	17.3	390		
rs4820268	TMPRSS6	AA	104.3	16.0	962	–0.74 (–1.52, 0.05) 0.07 3527	–0.23 (–1.23, 0.77) 0.66 2389
		AG	104.1	16.7	1837		
		GG	102.7	16.3	728		
rs1799945/rs1800562	2 risk alleles	0	106.1	16.3	156	0.11 (–0.83, 1.05) 0.82 3490	0.99 (–0.19, 2.18) 0.10 2312
	3 risk alleles	1	103.1	16.5	1163		
	4 risk alleles	2	104.0	16.4	2171		
genotypic score	⩽3 risk alleles	0	103.8	16.1	464	–0.32 (–0.94, 0.30) 0.31 3444	0.22 (–0.54, 0.98) 0.57 2254
	4 risk alleles	1	104.4	16.7	1143		
	5 risk alleles	2	103.5	16.1	1392		
	6 risk alleles	3	103.3	16.8	445		

a Effect=mean difference in offspring IQ per risk allele or unit increase in genetic score.

b Model adjusted by iron supplementation, child's genotype or score, maternal education and population stratification.

## Box 1 SNPs used in this study

*HFE* rs1800562 and rs1799945

Two mutations in the hemochromatosis (HFE) gene are known to cause iron overload (hemochromatosis) by affecting iron metabolism and storage (C282Y rs1800562 and H63D rs1799945).^19^

*TMPRSS6* rs4820268

Finberg *et al.*^23^ recently showed that mutations in the trans-membrane protease, serine 6 gene (TMPRSS6) could cause iron-deficiency anemia refractory to oral iron therapy. In genome wide association studies the synonymous variant rs4820268 in TMPRSS6 has been found to be associated with haemoglobin levels^22^ and serum iron levels.^20^

*TF* rs3811647

SNPs in the transferrin gene (TF), which codes for an iron carrying plasma protein and has previously been implicated in iron deficiency in pregnancy,^24^ were found to be strongly associated with serum transferrin levels with the strongest association for rs3811647.^20^
